# Emergency Department Visits for Pedestrians Injured in Motor Vehicle Traffic Crashes — United States, January 2021–December 2023

**DOI:** 10.15585/mmwr.mm7317a1

**Published:** 2024-05-02

**Authors:** Vaughn Barry, Miriam E. Van Dyke, Jasmine Y. Nakayama, Hatidza Zaganjor, Michael Sheppard, Zachary Stein, Lakshmi Radhakrishnan, Emily Schweninger, Kenneth Rose, Geoffrey P. Whitfield, Bethany West

**Affiliations:** ^1^Division of Injury Prevention, National Center for Injury Prevention and Control, CDC; ^2^Division of Nutrition, Physical Activity, and Obesity, National Center for Chronic Disease and Health Promotion, CDC; ^3^Office of Public Health Data, Surveillance, and Technology, CDC; ^4^Office of Policy Coordination and Development in the Office of the Secretary, U.S. Department of Transportation, Washington, DC.

**Summary**

**What is already known about this topic?**

Traffic-related pedestrian injuries are preventable but are increasing in the United States. In 2021, approximately 7,000 pedestrians died in motor vehicle crashes, representing a 40-year high.

**What is added by this report?**

During January 2021–December 2023, the proportion of all emergency department visits for pedestrian injury was highest among six racial and ethnic minority groups, persons aged 15–34 years, and males and during September–November.

**What are the implications for public health practice?**

Timely pedestrian injury data can help collaborating federal, state, and local partners rapidly monitor trends, identify disparities, and implement strategies supporting the Safe System approach, a framework designed to protect all road users.

## Abstract

Traffic-related pedestrian deaths in the United States reached a 40-year high in 2021. Each year, pedestrians also suffer nonfatal traffic-related injuries requiring medical treatment. Near real-time emergency department visit data from CDC’s National Syndromic Surveillance Program during January 2021–December 2023 indicated that among approximately 301 million visits identified, 137,325 involved a pedestrian injury (overall visit proportion = 45.62 per 100,000 visits). The proportions of visits for pedestrian injury were 1.53–2.47 times as high among six racial and ethnic minority groups as that among non-Hispanic White persons. Compared with persons aged ≥65 years, proportions among those aged 15–24 and 25–34 years were 2.83 and 2.61 times as high, respectively. The visit proportion was 1.93 times as high among males as among females, and 1.21 times as high during September–November as during June–August. Timely pedestrian injury data can help collaborating federal, state, and local partners rapidly monitor trends, identify disparities, and implement strategies supporting the Safe System approach, a framework for preventing traffic injuries among all road users.

## Introduction

In 2021, approximately 7,000 pedestrians were killed in motor vehicle crashes, the most in 40 years (*1*). During 2009–2016, approximately 47,000 traffic-related hospital admissions occurred annually among pedestrians (*2*). Data commonly used to assess pedestrian injuries, such as nationally representative probability sampled surveys of hospitals and police crash reports, might have time lags of ≥2 years between data collection and availability because of time required for data collection, coding, and review.^†^ Data timeliness is increasingly important to rapidly identify emerging shifts in injury patterns and evaluate prevention policies, programs, practices, and funding efforts to reduce pedestrian injuries. This report details pedestrian injury data for January 2021–December 2023 from the National Syndromic Surveillance Program (NSSP), a source of near real-time emergency department (ED) data.

## Methods

### Data Source

CDC used NSSP^§^ data to examine ED visits for pedestrian injuries. NSSP is a collaboration among CDC, local and state health departments, and federal, academic, and private sector partners. The program receives electronic health record data from approximately 78% of EDs nationwide, often within 24 hours.

### Definitions and Data Analysis

Traffic-related pedestrian injury ED visits (pedestrian visits) were initial encounters (i.e., not follow-up visits) for pedestrians unintentionally injured in motor vehicle crashes on public roads during January 3, 2021–December 31, 2023. Pedestrian visits were identified using a combination of administrative diagnosis codes and free-text reason-for-visit terms, developed and validated by CDC in partnership with five state or local health departments.^¶^ The pedestrian visit proportion (visit proportion), defined as the number of pedestrian visits per 100,000 total ED visits, was calculated overall and by race and ethnicity,** age group (0–14, 15–24, 25–34, 35–64, and ≥65 years), sex (female and male), season (autumn [September–November], winter [December–February], spring [March–May], and summer [June–August]), and U.S. Department of Health and Human Services (HHS) region.^††^ Visit ratios, with corresponding Wald 95% CIs, were calculated as the visit proportion of a given group divided by the visit proportion of a specified referent group.^§§^ Because data quality and coding practices can vary by facility and over time, analyses were restricted to EDs that more consistently reported complete data (coefficient of variation ≤40% and average weekly informative discharge diagnosis ≥75% complete during 2021–2023). After this restriction, 81% of all ED visits and 82% of all pedestrian ED visits were used.^¶¶^ Analyses were conducted using Base R (version 4.2.2; Posit). This activity was reviewed by CDC, deemed not research, and was conducted consistent with applicable federal law and CDC policy.***

## Results

The weekly number of pedestrian visits during the 3-year period (January 2021–December 2023) generally peaked during autumn. Weekly pedestrian visits mostly followed the pattern of all ED visits, with the exception that pedestrian visits flattened during summer while all ED visits increased (Figure).

**FIGURE:**
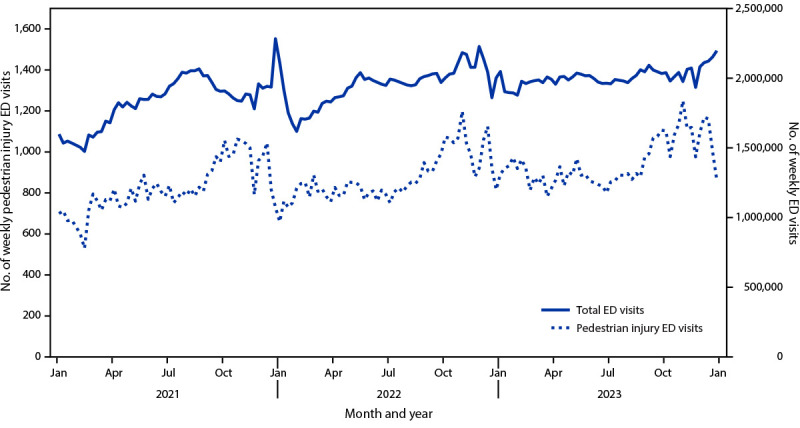
Weekly number of emergency department visits for pedestrian injury* — National Syndromic Surveillance Program,^†^ United States, January 2021–December 2023 **Abbreviations**: ED = emergency department; NSSP = National Syndromic Surveillance Program. * ED visits for an initial pedestrian injury encounter were identified by querying a categorization developed and validated by CDC in partnership with state and local health departments. This categorization aims to detect initial ED visits among pedestrians unintentionally injured on public roads in crashes involving a motor vehicle. https://knowledgerepository.syndromicsurveillance.org/sites/default/files/2023-10/CDC%20Pedestrian%20Motor%20Vehicle%20Traffic%20Injury%20v1.pdf ^†^ NSSP is a collaboration among CDC, federal partners, local and state health departments, and academic and private sector partners. NSSP receives medical record data from approximately 78% of EDs nationwide, although <50% of facilities from California, Hawaii, Minnesota, and Oklahoma currently participate in NSSP. https://www.cdc.gov/nssp/index.html

Among approximately 301 million ED visits, 137,325 involved a pedestrian injury, resulting in an overall visit proportion of 45.62 pedestrian injury ED visits per 100,000 total ED visits. Compared with the visit proportion among non-Hispanic White (White) persons, visit proportions were 2.47 times as high among non-Hispanic multiracial persons or persons of another race, 2.23 times as high among non-Hispanic Asian (Asian) persons, 2.13 times as high among non-Hispanic American Indian or Alaska Native (AI/AN) persons, 1.93 times as high among non-Hispanic Black or African American (Black) persons, 1.70 times as high among Hispanic or Latino (Hispanic) persons, and 1.53 times as high among non-Hispanic Native Hawaiian or Pacific Islander persons (Table). Compared with the visit proportion among persons aged ≥65 years, visit proportions were 2.83 times as high among persons aged 15–24 years, 2.61 times as high among those aged 25–34 years, 2.18 times as high among those aged 35–64 years, and 1.25 times as high among those aged 0–14 years. The visit proportion was 1.93 times as high among males as among females. Compared with visit proportions during summer, the visit proportion was highest during autumn (visit ratio = 1.21). Compared with visit proportion in HHS Region 7 (Iowa, Kansas, Missouri, and Nebraska), the visit proportion was 4.29 times as high in HHS Region 2 (New Jersey and New York).

**TABLE:** Emergency department visits for pedestrian injury* per 100,000 total visits and visit ratios, by selected characteristics — National Syndromic Surveillance Program,^†^ United States, January 2021–December 2023

Characteristic^§^	Visit proportion^¶^	Visit ratio** (95% CI)
**Overall**	**45.62**	**—**
**Race and ethnicity** ^††^		
American Indian or Alaska Native	68.24	2.13 (2.07–2.19)
Asian	71.51	2.23 (2.19–2.27)
Black or African American	61.88	1.93 (1.91–1.95)
Native Hawaiian or Pacific Islander	49.09	1.53 (1.41–1.66)
White	32.06	Ref
Hispanic or Latino	54.37	1.70 (1.68–1.71)
Multiracial or another race	79.21	2.47 (2.44–2.50)
**Age group, yrs**		
0–14	29.50	1.25 (1.23–1.28)
15–24	66.67	2.83 (2.79–2.88)
25–34	61.46	2.61 (2.57–2.65)
35–64	51.38	2.18 (2.15–2.22)
≥65	23.53	Ref
**Sex**		
Female	31.93	Ref
Male	61.57	1.93 (1.91–1.94)
**Season**		
Sep–Nov	51.01	1.21 (1.20–1.22)
Dec–Feb	45.79	1.09 (1.08–1.10)
Mar–May	43.42	1.03 (1.02–1.04)
Jun–Aug	42.10	Ref
**HHS region** ^§§^		
1	44.14	1.83 (1.73–1.92)
2	103.56	4.29 (4.08–4.50)
3	43.14	1.78 (1.70–1.88)
4	37.26	1.54 (1.47–1.62)
5	33.28	1.38 (1.31–1.45)
6	38.87	1.61 (1.53–1.69)
7	24.17	Ref
8	44.24	1.83 (1.73–1.93)
9	51.45	2.13 (2.02–2.24)
10	43.28	1.79 (1.70–1.89)

**Abbreviations**: ED = emergency department; HHS = U.S. Department of Health and Human Services; NSSP = National Syndromic Surveillance Program; Ref = referent group.

* ED visits for an initial pedestrian injury encounter were identified by querying a categorization developed and validated by CDC in partnership with state and local health departments. This categorization aims to detect initial ED visits among pedestrians unintentionally injured on public roads in crashes involving a motor vehicle. https://knowledgerepository.syndromicsurveillance.org/sites/default/files/2023-10/CDC%20Pedestrian%20Motor%20Vehicle%20Traffic%20Injury%20v1.pdf

^†^ NSSP is a collaboration among CDC, federal partners, local and state health departments, and academic and private sector partners. NSSP receives medical record data from approximately 78% of EDs nationwide, although <50% of facilities from California, Hawaii, Minnesota, and Oklahoma currently participate in NSSP. https://www.cdc.gov/nssp/index.html

^§^ Percentage of missing data for total ED visits and for pedestrian injury ED visits was sex <1% and <1%, respectively; age <1% and 2%, respectively; race and ethnicity 8% and 8%, respectively; season and HHS region 0% and 0%, respectively.

^¶^ (Number of ED visits for pedestrian injury / total number of ED visits) x 100,000.

** (ED visits for pedestrian injury [comparison group] / all ED visits [comparison group]) / (ED visits for pedestrian injury [Ref] / all ED visits [Ref]).

^††^ Patients missing ethnicity were categorized as non-Hispanic and based on documented race. Patients missing race data and who were not documented as Hispanic or Latino (Hispanic) were categorized as missing. Persons of Hispanic origin might be of any race but are categorized as Hispanic; all racial groups are non-Hispanic.

^§§^ https://www.hhs.gov/about/agencies/iea/regional-offices/index.html; https://www.cdc.gov/nssp/participation-coverage-map.html

## Discussion

Using syndromic surveillance data from January 2021–December 2023, the proportion of ED visits related to pedestrian injury was highest among six racial and ethnic minority groups. The racial and ethnic disparities in this report are consistent with previous studies. For example, among patients in the U.S. Nationwide Inpatient Sample during 2009–2016, admission rates were elevated among Black, Hispanic, and multiracial persons and persons of another race (*2*). Pedestrian death rates nationwide during 2018 were higher among AI/AN and Black persons than among White persons (*3*). However, the visit proportion in the current study was higher among Asian persons than among White persons, whereas pedestrian death rates in 2018 indicated the reverse (*3*).

Unsafe walking environments and limited investment in infrastructure for pedestrians (e.g., sidewalks, street lighting, and crosswalks) can result from past development that prioritized vehicles (*4*) and historical segregation and disinvestment in neighborhoods based on race and income (*5*). Healthy community design strategies exist that address pedestrian injury inequities while minimizing harms, such as displacement, that can occur among persons from some racial and ethnic groups and with lower incomes (*6*).

In addition to racial and ethnic inequities, differences were also found by sex, age, season, and region. The higher proportion of pedestrian visits among males aligns with 2021 pedestrian death rates (*1*). The visit proportion was highest among persons aged 15–24 years compared with other age groups. This finding differs from 2021 pedestrian death rates, which were highest among adults aged 60–64 years (*1*), likely because of increasing frailty with age (*7*). The pedestrian visit proportion was highest during autumn, as was the number of pedestrian deaths in traffic crashes during 2020–2021.^†††^ Variation in regional visit proportions might be influenced by differences in pedestrian volume or population density.^§§§^

Risk factors for pedestrian injury are generally multifactorial and can include exposure to vehicles traveling at high speeds, alcohol involvement on the part of the driver or pedestrian, and insufficient visibility. Slowing vehicles by narrowing or reducing lanes, reducing speed limits, or using automated speed cameras can protect pedestrians, as can improving crossing safety and separating pedestrians from vehicles through new or improved sidewalks (*8*,*9*). In 2021, an estimated 19% of crashes resulting in pedestrian deaths involved drivers with blood alcohol concentrations of ≥0.08 g/dL (*1*). Despite proven effectiveness of stricter blood alcohol limits (*9*), only one state, Utah, has lowered its legal blood alcohol concentration from 0.08 to 0.05 g/dL. In the year after the law went into effect, the motor vehicle crash death rate per mile driven decreased 18% in Utah, compared with a 6% decrease in the rest of the United States (*10*). Most pedestrian traffic deaths (77% in 2021) occurred after dark (*1*). Enhancing visibility through strategies such as street lighting can help reduce pedestrian traffic deaths.

A comprehensive approach involving collaboration among federal, state, and local partners could help prevent pedestrian injuries and address social and structural inequities that contribute to traffic-related injury risk. The Safe System approach^¶¶¶^ provides a framework for helping prevent traffic injuries among all road users and minimizing harm when injuries occur and is based on five core elements: safer people, safer roads, safer speeds, safer vehicles, and better postcrash care. An example of collaboration within the Safe System approach is coordination between state and local communities on speed management strategies. Although decisions about road speeds are usually controlled at the state level, local communities increasingly recognize the importance of managing vehicle speed for pedestrian safety. Timely ED data on pedestrian injuries could contribute to state and local data-driven safety traffic plans that help guide similar collaborative prevention strategies to create safer pedestrian environments. The Road to Zero Coalition**** has assembled organizations and federal partners to work together to achieve zero crash deaths by 2050, using strategies that adopt the Safe System approach. Partners include CDC and the U.S. Department of Transportation. The National Roadway Safety Strategy,^††††^ released in 2022, outlines the U.S. Department of Transportation’s strategy, emphasizing the Safe System approach. The 2021 Infrastructure Investment and Jobs Act^§§§§^ provided funding for transportation programs designed to reduce injury risk and disparities among pedestrians.

### Limitations

The findings in this report are subject to at least five limitations. First, NSSP data are not nationally representative. Second, this report includes only a percentage of U.S. EDs, and causes of injuries are not always documented in medical records; therefore, the weekly numbers of pedestrian injury ED visits are likely underestimates. Third, EDs might collect race and ethnicity data differently, which could result in misclassification. Fourth, detailed crash information such as vehicle speed, time of day, roadway and pedestrian infrastructure, and driver and pedestrian behavior (e.g., impairment) are not available in NSSP. Finally, differences in ED usage across groups, both general usage and that specific to pedestrian injuries, could affect results.

### Implications for Public Health Practice

Findings from ED data on pedestrian injuries emphasize the need to prioritize prevention efforts for pedestrians. NSSP provides near real-time pedestrian injury data. These data can be analyzed at the local, state, and national levels to monitor the most recent trends, identify populations and areas most affected, and tailor implementation strategies supporting the Safe System approach, a framework designed to protect all road users.

## Footnotes

* These authors contributed equally to this report.

† Crash Report Sampling System (https://www.nhtsa.gov/crash-data-systems/crash-report-sampling-system); Fatality Analysis Reporting System (https://www.nhtsa.gov/research-data/fatality-analysis-reporting-system-fars); Healthcare Cost and Utilization Project (https://hcup-us.ahrq.gov/databases.jsp); National Electronic Injury Surveillance System-All Injury Program data (https://health.gov/healthypeople/objectives-and-data/data-sources-and-methods/data-sources/national-electronic-injury-surveillance-system-all-injury-program-neiss-aip); and National Hospital Ambulatory Medical Care Survey (https://www.cdc.gov/nchs/ahcd/datasets_documentation_related.htm).

§
https://www.cdc.gov/nssp/index.html

¶ A pedestrian is defined as any person on foot, walking, running, jogging, sitting or lying down, in a motorized or nonmotorized wheelchair, in a baby carriage, on roller skates or inline skates, on a skateboard, on a nonmotorized scooter, on a motorized mobility scooter designed to accommodate disability, or on skis, sleds, or ice skates. To identify *International Classification of Diseases, Ninth Revision, Clinical Modification* and *International Classification of Diseases, Tenth Revision, Clinical Modification* codes for pedestrian motor vehicle traffic injuries, codes currently used or recommended for pedestrian injury surveillance were reviewed and considered for inclusion. These included (but were not limited to) codes used in surveillance at the National Highway Traffic Safety Administration, at CDC, and the codes recommended in the Consensus Recommendations for Pedestrian Injury Surveillance by the Safe States Alliance. https://knowledgerepository.syndromicsurveillance.org/sites/default/files/2023-10/CDC%20Pedestrian%20Motor%20Vehicle%20Traffic%20Injury%20v1.pdf

** Race and ethnicity are categorized as non-Hispanic White, non-Hispanic Black or African American, non-Hispanic American Indian or Alaska Native, non-Hispanic Native Hawaiian or Pacific Islander, non-Hispanic Asian, non-Hispanic multiracial or another race, and Hispanic or Latino.

†† https://www.hhs.gov/about/agencies/iea/regional-offices/index.html; https://www.cdc.gov/nssp/participation-coverage-map.html

§§ Referent groups are defined as the groups with the lowest proportions for pedestrian injury ED visits per 100,000 visits.

¶¶
https://www.cdc.gov/nssp/dqc/articles/how-data-quality-filters-work.html

*** 45 C.F.R. part 46.102(l)(2), 21 C.F.R. part 56; 42 U.S.C. Sect. 241(d); 5 U.S.C. Sect. 552a; 44 U.S.C. Sect. 3501 et seq.

†††
https://www.cdc.gov/injury/wisqars/fatal/trends.html

§§§
https://journals.sagepub.com/doi/10.1177/0739456X19845043

¶¶¶
https://publichealth.jhu.edu/sites/default/files/2023-03/recommendations-of-the-safe-system-consortium.pdf

****
https://www.nsc.org/roadtozero

††††
https://www.transportation.gov/NRSS

§§§§
https://www.congress.gov/bill/117th-congress/house-bill/3684/text
